# Qualitative study on the implementation of professional pharmacy services in Australian community pharmacies using framework analysis

**DOI:** 10.1186/s12913-016-1689-7

**Published:** 2016-08-25

**Authors:** Joanna C. Moullin, Daniel Sabater-Hernández, Shalom I. Benrimoj

**Affiliations:** 1 Graduate School of Health, University of Technology Sydney, Level 4, Building 7, 67 Thomas St Ultimo, (PO Box 123 Broadway), Ultimo, 2007 NSW Australia; 2 Academic Centre in Pharmaceutical Care, Pharmaceutical Care Research Group, Faculty of Pharmacy, University of Granada, Campus Universitario de Cartuja s/n. C.P. 18071, Granada, Spain

**Keywords:** Health services [MeSH], Health plan implementation [MeSH], Communication [MeSH], Health services research [MeSH], Health services administration [MeSH], Pharmaceutical services [MeSH]

## Abstract

**Background:**

Multiple studies have explored the implementation process and influences, however it appears there is no study investigating these influences across the stages of implementation. Community pharmacy is attempting to implement professional services (pharmaceutical care and other health services). The use of implementation theory may assist the achievement of widespread provision, support and integration. The objective was to investigate professional service implementation in community pharmacy to contextualise and advance the concepts of a generic implementation framework previously published.

**Methods:**

Purposeful sampling was used to investigate implementation across a range of levels of implementation in community pharmacies in Australia. Twenty-five semi-structured interviews were conducted and analysed using a framework methodology. Data was charted using implementation stages as overarching themes and each stage was thematically analysed, to investigate the implementation process, the influences and their relationships. Secondary analyses were performed of the factors (barriers and facilitators) using an adapted version of the Consolidated Framework for Implementation Research (CFIR), and implementation strategies and interventions, using the Expert Recommendations for Implementing Change (ERIC) discrete implementation strategy compilation.

**Results:**

Six stages emerged, labelled as development or discovery, exploration, preparation, testing, operation and sustainability. Within the stages, a range of implementation activities/steps and five overarching influences (pharmacys' direction and impetus, internal communication, staffing, community fit and support) were identified. The stages and activities were not applied strictly in a linear fashion. There was a trend towards the greater the number of activities considered, the greater the apparent integration into the pharmacy organization. Implementation factors varied over the implementation stages, and additional factors were added to the CFIR list and definitions modified/contextualised for pharmacy. Implementation strategies employed by pharmacies varied widely. Evaluations were lacking.

**Conclusions:**

The process of implementation and five overarching influences of professional services implementation in community pharmacy have been outlined. Framework analysis revealed, outside of the five overarching influences, factors influencing implementation varied across the implementation stages. It is proposed at each stage, for each domain, the factors, strategies and evaluations should be considered. The Framework for the Implementation of Services in Pharmacy incorporates the contextualisation of implementation science for pharmacy.

**Electronic supplementary material:**

The online version of this article (doi:10.1186/s12913-016-1689-7) contains supplementary material, which is available to authorized users.

## Background

Implementation research is evolving, but studies using implementation theory and investigation of the implementation influences, over the course of implementation during each implementation stage, is scarce in both pharmacy and other disciplines [1, 2]. Knowledge of pharmacy’s implementation process, combined with the use of a suitable implementation framework(s), could aid widespread adoption, implementation, sustainability and eventual scale-up of professional pharmacy services. Correspondingly as professional pharmacy services incorporate the principles and practices of pharmaceutical care and clinical pharmacy [3, 4] an improvement in patient outcomes would be predicted. Examples of professional pharmacy services include conducting reviews of patients’ medications, counselling on new and/or chronic medications (to improve health literacy, knowledge, adherence and prescribing behaviour), the provision of immunisations and involvement in public health promotional campaigns [3, 5, 6]. In Australia these services may be Government funded, such as medication reviews, which may be adopted by any pharmacy, introduced by pharmacy groups, which may be optional or ‘mandatory’ for branches to provide, or developed and introduced at individual pharmacy level.

Internationally community pharmacy is attempting to implement professional services into routine practice [7–9]. In several countries professional pharmacy services are being remunerated and pharmacies are beginning to implement, however the implementation process pharmacies are undergoing is largely unknown [10]. Pharmacy practice research remains predominantly focused on clinical and cost effectiveness of the professional services [11], barriers and facilitators [12–15], pharmacy culture [16], perception of pharmacy [10, 17], and remuneration [18, 19].

There is increasing consensus in implementation science and knowledge translation regarding the concepts involved in implementation [20]. Implementation is the process of commencing to use and integrating innovations within a setting [21]. This process is described as a non-linear, iterative and complex that may be divided into a number of stages [22–24] and activities/steps [25, 26]. Throughout each stage of the implementation process, three fundamental elements or influences should be considered: factors, strategies and evaluations. Specifically those wishing to implement should consider the factors that are influencing the implementation effort (also termed determinants of practice or barriers and facilitators) [27–30], which strategies may assist (including implementation interventions) [30–35] and what evaluations should be conducted (encompassing tools, measures and outcomes) [36–40]. Finally the constituents within the factors, strategies and evaluations may be grouped into contextual domains or ecological levels [27, 41]. In other words, factors exist at multiple levels and strategies and evaluations should be targeted towards each level. In brief, implementation may be summarised as involving: (1) an innovation, and (2) a process, influenced across (3) contextual domains/levels by (4) factors (5) strategies (6) and evaluations [20]. There are a range of frameworks, models or theories that target the concepts individually as well as holistically [Table 1] [20, 42]. See Additional file 1 for implementation definitions.

**Table 1:** Examples of meta-frameworks and models

Concept	Framework examples
Process	
Stages	Greenhalgh et al. [22], Fixsen et al. [24], Aarons et al. [23]
Steps	Meyers et al. [25, 26]
Domains	Greenhalgh et al. [22], Damschroder et al. [27], Wandersman et al. [41]
Factors	Damschroder et al. [27], Flottorp et al. [28], Michie et al. [29, 30]
Strategies	
Discrete	EPOC [31], Mazza et al. [32], Powell et al. [33, 34], Michie [30, 35]
Multifaceted	Glisson et al. [66], Chinman et al. [52], Kilbourne et al. [53], Institute for Healthcare Improvement [67, 68]
Evaluations	Proctor et al. [37], Glasgow et al. [36], Steckler et al. [39], Lehman et al. [38], Stetler et al. [40] Green et al. [69]

**Fig. 1:**
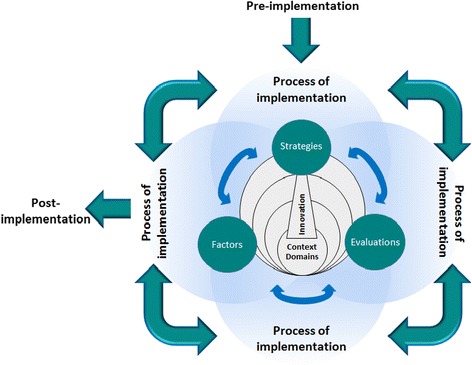
Generic Implementation Framework (GIF)

The Generic Implementation Framework (GIF) has been suggested as an overarching, broad framework that collates and illustrates the core implementation concepts, suitable across disciplines [Fig. 1] [20]. The GIF is a skeletal structure into which specific, detailed meta-frameworks, models or theories, such as those detailed in Table 1, should be chosen for each concept: innovation, process, contextual domains, factors, strategies and evaluations. To tailor the GIF to pharmacy practice it is therefore necessary to investigate and determine the contents for each of the aforementioned implementation concepts.

### Aim of study

The general objective was to explore the concepts of the GIF in community pharmacy in order to tailor a framework for the implementation of services in pharmacy. The primary objective was to investigate the process of professional service implementation occurring in practice and secondarily to assess over the course of this process the factors, strategies and evaluations.

## Methods

Semi-structured interviews were conducted and analysed using framework methodology.

### Interview design

Face to face semi-structured interviews were chosen to enable a confidential exploration of pharmacists’ experiences, behaviours, practice, process and perceptions in the implementation of professional pharmacy services. An eight question interview guide was developed and used [Additional file 2]. The structure and questions of the interview guide were derived from a systematic review of implementation frameworks and the resulting GIF concepts [20]. The guide examined across the stages of exploration, preparation, operation and sustainability, the steps or activities pharmacies conducted, factors that influenced, strategies used and evaluations conducted, if any. The interview guide was piloted in two pharmacies to establish face validity. These pilot pharmacies were not included in the purposeful sampling process and therefore were not included in the analysis.

### Sampling and recruitment

Purposeful sampling was used to maximise variation in pharmacies’ level of service implementation across three states of Australia. De-identified data from a pharmacy service software provider, servicing over 60 % of Australian pharmacies, was used to locate pharmacies that appeared to be at various stages of the implementation process, based on the number of MedsCheck services they were currently providing (<10, 11–100, 101–400 or >400 MedsChecks in the period twelve month period between December 2012 and November 2013). The number of MedsChecks was used to select pharmacies as it was a service introduced in 2012, remunerated, provided within community pharmacies and required distinct practice and organisational changes to be implemented. MedsChecks are basic medication review services, similar to Medicines Use Review (MUR) [43], that involve a consultation (estimated to be 30 to 40 min), by a registered pharmacist who is not undertaking other professional duties at the time, face to face with the patient, in an area of a community pharmacy that is physically separated from the trading floor to ensure privacy and confidentiality. Pharmacies had to be within a two hours’ drive from a capital city to be included in the study.

Pharmacies were contacted by the software provider to ask permission for the research team to communicate. Pharmacies who agreed to be contacted were recruited in December 2013 by phone and an interview time arranged with a consenting pharmacist. Information was offered to be emailed at this time and was given to all participants, along with signing a written consent in person, prior to the interview. Pharmacy owners, managers and employee pharmacists, involved in the implementation and/or provision of professional pharmacy services were interviewed [Participant demographics see Table 2]. In four pharmacies a second pharmacist was available and consented to being interviewed on the day.

**Table 2:** Participant characteristics

		Pharmacists *n* = 25	Pharmacies *n* = 21
Staff Type	Employee	9	
	Services Manager	2	
	Pharmacy manager	7	
	Owner	7	
State of Australia	NSW	17	13
	VIC	1	1
	WA	7	7
Number of MedsCheck Services^a^	<10	9	7
	11–100	7	6
	101–400	4	3
	>400	5	5
Pharmacy Size^b^	Small	6	5
	Small-medium	8	6
	Medium	6	6
	Large	5	4
Pharmacy Type^c^	Independent	6	5
	Banner group	12	11
	Discount chain	7	5
Pharmacy Location	Local shopping strip	12	10
	Central Business District	4	3
	Shopping centre (mall)	9	8

^a^Provided for the year between December 2012 – and November 2013

^b^Size determined by number of pharmacists on duty majority of the time: Small = 1, Small-medium = 2, Medium = 3–4, Large = ≥5

^c^Banner groups: pharmacies who act as a franchise for marketing, management and purchasing purposes Discount chains: banner groups marketed as discounters

### Setting, data collection and data management

Interviews were conducted within a quiet area of the community pharmacies between January and February 2014. All interviews were audiotaped and subsequently transcribed in full and managed by QRS nVIVO 10.

### Data analysis

A constructivist qualitative methodology, framework analysis, was used to analyse the data [44–46]. Framework analysis allowed for assessment of the data both across the interview cases and within the stages. The first phase of the framework methodology was familiarisation of the raw data by listening to audiotapes, to confirm accuracy of transcripts, and to note key ideas and recurrent themes. The data was then coded under the stages of implementation as the overarching themes, according to definitions in Additional file 1, and charted into a framework matrix. Charting is where data is rearranged and summarized, each column being a theme (stages of implementation) and each row a case (pharmacists interviewed) [44], to facilitate a detailed view of the implementation stages and the constituents within each stage for each interview case.

Thematic analysis was performed on the data under each stage of implementation to identify the steps/activities and influences on the process [44, 45]. This analysis was performed by open coding the transcript line-by-line, using a constant comparison approach of coding and recoding the interviews, until each pharmacists’ interview data was coded across all applicable implementation stages and the key activities and influences in the implementation process emerged. Additional codes were added as the data extraction continued allowing the framework to be developed further [45]. The interpretation of the chart was used to confirm the implementation process, the influences and their relationships [44, 45].

A basic secondary analysis was performed to examine the influences using established implementation frameworks of the elements (factors, strategies and evaluations) across the domain levels [Additional file 1]. Specific implementation frameworks, which fit within the overarching concepts of the GIF, were used to structure the analysis. Instead of the largely inductive thematic analysis performed of the influences within the framework matrix, a more deductive approach was utilised to further investigate and advance the frameworks. Factors were assessed at each stage of implementation using the Consolidated Framework for Implementation Research (CFIR) [27]. CFIR was augmented with factors not included, or implied within broad constructs of the framework, in order to make them more explicit. Additional factors included behavioural influences from Theoretical Domains Framework and Behavioural Change Wheel [29, 47], and previous pharmacy practice research, such as remuneration [48]. Other adaptations included dividing the outer setting into two, the “external system” (economic, political and professional milieu) and “local environment” (circumstances surrounding the organisation (s) including patient, community, network) and the inner setting was termed “organisation” and intervention called “innovation” for greater clarity. These changes were based on implementation literature [49, 50] assessments of CFIR [51], and pharmacy practice literature [48]. The list of factors was further expanded with those that emerged from the interview data. Terminology was kept as consistent as possible to enable future comparative studies to be conducted. The strategies utilised by pharmacies were considered using with the more detailed Expert Recommendations for Implementing Change (ERIC) discrete implementation strategy compilation [34], rather than the general “process” construct of CFIR. As an initial analysis factors or strategies were marked in the analysis if they appeared in the interview data, thematic saturation and the degree of influence were not assessed. No further investigation of evaluations concept was conducted.

### Ethics, consent and permissions

This study was approved by University of Technology Sydney Ethics Committee (UTS HREC REF NO. 2013000670). A written consent of interviewees was obtained in person, prior to the interview.

## Results

Out of the 28 community pharmacies invited by the research team, 21 agreed to participate, with 25 interviews taking place. At this point thematic saturation of activities appeared to have been achieved, with no new activities emerging for any implementation stage, and therefore no further sampling was conducted [44]. Interviews ranged from 20 to 50 min. Participant characteristics are provided in Table 2. There was a range of levels of service provision, however all pharmacies had provided at least one service. The pharmacists interviewed spoke primarily about MedsCheck, but also the implementation of a range of other services including clinical interventions, sleep apnoea, health promotions (e.g. clean and check days for blood glucose monitors, community health talks, stroke prevention campaigns, flu vaccination), health screening or monitoring (blood pressure, blood glucose, cholesterol, iron, hearing), adherence, new to therapy, opioid replacement and mental health services. All pharmacies were conducting or considering at least one other service.

### Process of implementation

Six implementation stages emerged from the data, the four stages of the interview guide (exploration, preparation, operation and sustainability) and a further two stages. The additional stages were a pre-implementation stage of development or discovery and a testing stage prior to operation. Pharmacies also spoke of a range of implementation activities they completed as they moved through the stages [results presented in Table 3]. Quotes supporting the stages and activities of the implementation process are provided in Additional file 3. Analysis across the cases of the framework matrix revealed a trend towards the greater the number of activities considered, the greater the apparent integration into the pharmacy organization. In addition the interpretation of the framework matrix highlighted that there were overlaps between stages, variation in duration of stages, movement back and forth between stages, differences in the order of performing implementation activities and that not all activities were necessarily completed. For this reason, to appear less linear, the term activities was chosen rather than steps.

**Table 3:** Resulting stages and activities of the implementation process of professional pharmacy services in community pharmacy

Stages and activities of the implementation process of professional pharmacy services in community pharmacy	
Development or Discovery	
Exploration	
- Organisational fit assessment	
- Value assessment (relative advantage)	
- Service assessment (service characteristics)	
- Organisational capacity assessment (supporting conditions & staff capacity)	
- Community fit assessment	
- Decision	
Preparation	
- Assign leader	
- Research requirements	
- Organise supporting conditions	
- Plan service procedure	
- Rearrange workflow	
- Staff arrangements	
- Team communication (buy-in and foster climate)	
- Training	
- Community awareness & recruitment	
Testing	
- Initial adaptations	
- Familiarisation & improve staff conviction	
- Test patient demand	
Operation	
- Modification of plans & procedures	
- Maintain patient demand	
- Staffing	
- Teamwork, team input and internal communication	
- Integration tactics	
- Ongoing training	
- Goal setting	
- Monitoring	
- Adaptation	
- Improvement	
Sustainability	
- Monitoring^a^	
- Adaptation^a^	
- Improvement^a^	

^a^Few pharmacies had reached sustainability, these activities appeared in the few that had continued service delivery after funding changes, however require further assessment

A trigger was often involved to move pharmacies into the stages of development or exploration. Triggers included a new employee (generally at managerial level), financial stress, pressure from the pharmacy group, attendance of a workshop or conference and/or a representative visit from a pharmaceutical company or software provider.

#### Development or discovery

A pre-implementation stage emerged in the discourse where a pharmacy or pharmacy group had to develop services within their pharmacy or group of pharmacies, and/or discover externally developed services. Services developed internally were primarily testing (screening or monitoring) or health promotions. The majority of pharmacists appeared to hear about an externally developed service (company sponsored and government programs), from the Pharmacy Guild of Australia (membership body), through internal group communications (if part of a pharmacy group), or through a personal initiative, such as speaking to colleagues or attending a conference. Poor communication and awareness of government programs was an issue raised by pharmacists.

#### Exploration

During the exploration stage the service was assessed to see if was aligned with the pharmacy’s orientation [Activity: *organisational fit assessment* [See Table 3 for activities and Additional file 3 for quotations]]. Pharmacies also looked at the potential benefits the service would offer, including financial, business (such as increasing customer loyalty and rapport), patient and/or professional [activity: *value assessment (relative advantage)*]. Service benefits were balanced against the ‘implementability’ and ‘workability’ of the service in most cases. That is pharmacies assessed the service itself (duration of service and follow-up, degree of change etc.) [activity: *service assessment (service characteristics)*] and their capacity (cost of resources, staffing levels, training etc.) [activity: *organisational capacity assessment (supporting conditions & staff capacity)*]. Some pharmacies considered their community’s needs, demographics, rapport and estimated demand based on how they believed their patients’ perceive pharmacy and would perceive the service [activity: *community fit assessment*].

The exploration or appraisal stage was often informal, without set structure or systems, but a few of pharmacies did a more formal, objective assessment. A decision was subsequently made, by the owner or the owner in consultation with senior pharmacist (s)/manager, to adopt or reject the service [activity: *decision*].

#### Preparation

After deciding to adopt a service in many cases a staff member was assigned to be in charge of the service, informally or formally, explicitly or implicitly [activity: *assign leader*]. This person was most often a pharmacist employee, but also included the owner, pharmacy technician or pharmacy assistant. Some pharmacies had one staff member in charge across multiple services, while other pharmacies delegated different employees to particular services. The leader’s tasks included conducting training, recruiting patients, providing the service and overall driving the implementation effort. Another activity was to investigate the legalities and necessities of the service [activity: *research requirements*] and making the required changes to ensure the conditions were satisfactory [activity: *organise supporting conditions*].

Planning a procedure of how to deliver the service was generally carried out by the leader of the service [activity: *plan service procedure*]. All pharmacies considered logistics, but this was a particularly significant activity for smaller pharmacies with few staff or those with acutely busy periods of the day, such as those working in the city centre. As part of procedure planning some pharmacies developed an individualised protocol for the delivery of the service, while others relied on external guidelines, support provided by their pharmacy group, or support from an external body such as a pharmaceutical company. Along with the procedure of the specific service, for some pharmacies, preparation involved considering the workflow of the dispensary or the whole pharmacy [activity: *rearrange workflow*]. As an example one pharmacy moved a pharmacist to the front counter to interact with patients and hand-out prescriptions rather than dispense.

Staffing was a major consideration including changing staff roles and responsibilities, analysing staff numbers (to facilitate provision and meet regulatory requirements) and staff selection if new staff were required [activity: *staff arrangements*]. There was wide variability in the level of team input and teamwork [activity: *team communication (buy-in and foster climate)*]. Internal communication channels were fairly equally spread between formal meetings, informal conversations or lacking altogether.

Training was one of the most quoted activities undertaken to prepare for service delivery [activity: *training*]. While another consideration were methods to increase community awareness and commence patient recruitment [activity: *community awareness & recruitment*]. Both activities were led by the individual pharmacy, the pharmacy group, and/or supported by an external party.

#### Testing

A few pharmacies showed a distinct stage where they were trialling the service, operating for a defined period or with limited numbers. The testing or initial operation stage was about refinement of procedures [activity: *initial adaptations*], familiarisation of the procedures and software, to increase staff members’ confidence, comfort and conviction with their role in the service [activity: *familiarisation & improve staff conviction*], and trialling the fit of service to the community in terms of patient perception and demand [activity: *test patient demand*].

#### Operation

As pharmacies moved to providing the service, procedures were further refined, including the protocol, logistics, recruitment process and/or data management system (for example if the computer programs were inadequate moving from computer, to iPad, to paper) [activity: *modification of plans & procedures]*. Service provision involved the new task of recruiting and enrolling patients and the implementation activity of maintaining patient demand emerged as a critical theme [activity: *maintain patient demand*]. The activity was approached in a number of ways including revising the dispensary procedure to include identifying patients, developing a uniform approach for asking patients, delegating to a staff member, using reminders and organising mail-outs. Most pharmacies had regular patients who they were able to enrol, however after this initial recruitment, most pharmacies struggled to maintain patient demand.

Staffing issues were deliberated by all pharmacies [activity: *staffing*]. Increasing staff skills and confidence, in providing the service and in the recruitment/selling of the service, as well as redefining roles and responsibilities of the pharmacy team were considered [activity: *teamwork team input and internal communication*]. Most pharmacies initiated techniques to assist breaking habits and to improve the integration of the service into routine practice [activity: *integration tactics*]. Tactics included reminders, providing incentives or disincentives and conducting performance reviews. In addition ongoing training for staff members was raised but was absent in the majority of cases [activity: *ongoing training]*.

Goal setting was prevalent in pharmacies more progressed in implementation [activity: *goal setting*]. A small number of pharmacies believed goals took away from the purpose of the service or that self-motivation was sufficient, some developed Key Performance Indicators (KPIs) for individual staff members, while others set pharmacy team targets. Goals were always based on number of patients or services provided.

Formal monitoring systems to record number of patients only emerged in a few pharmacies where it was organised by their pharmacy group [activity: *monitoring]*. Occasionally this was linked to pharmacy finances. The monitoring of customer feedback was seen as important to improve implementation and service provision as well to judge the relative advantage of the service. In addition there was informal monitoring of service procedures, such as time to conduct the service. Based on the monitoring a few pharmacies adapted the service, such as moving the location or time of the service, so it was done immediately rather than using an appointment system [activity: *adaptation*]. The final activity of operation was minor adjustments or improvements that were made to increase efficiency and proficiency, without changing the service [activity: *improvement*].

#### Sustainability

Few pharmacies had reached sustainability, that is ongoing service provision, maintenance of supportive conditions and persistence of service outcomes. Services were sustained only in those pharmacies that were able to adjust the service sufficiently to overcome changes in funding to maintain financial profitability and/or experienced relative advantage of the service in aspects other than from a financial perspective. An example of an adjustment was a service that had been reinvented from a government program to a private fee-for-service.

### Implementation influences

Five influences recurred in the thematic analysis of the implementation process: direction and impetus, internal communication, community fit, staffing and support. These influences affected a number of stages and activities, both positively and negatively, depending on their presence. For example increased staff capacity was positively associated to pharmacies progressing through implementation, whilst insufficient staff had negative effects on the adoption of change.

The importance of the pharmacy’s direction and impetus, which includes both the pharmacy’s vision and the top level leadership provided by the owner or manager, was a the first influence to emerge. A shift or change in a pharmacy’s vision, if this was not the existing orientation, was frequently the first requirement for the implementation process. This appeared to be an overarching prerequisite for further implementation. Top level leadership needed to include support, drive and push from the owner and/or manager. This type of leadership was necessary in addition to the role and responsibilities of an internal leader or champion.

The second influence was the internal communication within the pharmacy, including team-input and teamwork. Pharmacies ranged from having almost no communication surrounding services to formal buy-in and input of all staff throughout the process. Internal communication affected the pharmacy culture, implementation climate and subsequently the overall implementation effort.

The third influence was staff. Staff capacity (manpower, skills, and confidence) was particularly linked with the assessment decision during the exploration stage, but influenced all stages. Selecting staff and staff members’ beliefs regarding the innovation were major influences. For example pharmacists who saw services as something they already provided or did not see their value, appeared to struggle with implementation.

Community fit influenced all stages of the implementation process. Initially in exploration, the community’s demographics (patient needs and resources) was considered by a few pharmacies. As pharmacies moved through the implementation process the number of pharmacies thinking about community fit increased, as they became aware of the influence of community awareness, perception and demand.

The final overarching influence was support, which included having a professional network, pharmacy group support and/or external support. This support affected a number of implementation activities including establishing favourable conditions, developing a service procedure, training, goal setting, monitoring and adaptations.

### Secondary analysis

As a result of a secondary analysis of the data a refined list of implementation factors for community pharmacy was developed [Additional file 4]. In total seventeen additional factors were added to the CFIR, eleven factors derived from implementation and pharmacy practice literature and six from the interview analysis. The domains, as previously defined in a systematic review of implementation frameworks [20], were endorsed by the factors fitting within its structural arrangement. Factors varied across the implementation stages. The initial analysis of factors at each stage of implementation is provided in Additional file 5. Not surprisingly factors relating the characteristics service to be implemented (innovation domain) were particularly prominent during exploration, when pharmacies were deciding whether or not to adopt. Beliefs about the service (such as pharmacists not seeing value in it, or that it was a task they already performed and implementation was just documenting the task), staff personalities and self-efficacy were prominent factors relating to staff (individual factor domain), and could have both a positive and negative influence on the implementation process. All factors related to the pharmacy (organisational domain) were implicated during the operation stage, but the majority also during all stages. Quality assurance and data management systems were widely lacking. As mentioned a pharmacy’s patient population, their needs and subsequently the demand for the service were dominant community factors. Furthermore during development and exploration stages peer pressures from other pharmacies, either mimetic or competitive to differentiate, were factors. For factors relating to the external system (political, economic and regulatory environment) the funding model, political stability and external support by professional bodies and companies were the most pronounced and predominant influence on sustainability.

Implementation strategies employed by pharmacies to aid adoption and integration varied widely. Many pharmacies were struggling with implementation, yet out the 73 discrete implementation strategies described by Powell et al. [34], 51 were implicated by at least one pharmacy [Additional file 6]. Despite the large number of strategies used, generally only one or two pharmacies utilised any one strategy.

During the framework analysis,  evaluations of any form, were shown to be generally lacking or informal and therefore no secondary analysis was performed. All pharmacies looked at numbers of services provided (sometimes linking to economic outcomes) and patient feedback was used to gauge service, humanistic outcomes. There appeared to be no performance, implementation or clinical evaluations.

## Discussion

Pharmacies in Australia, appeared to pass through stages of implementation and completed many implementation activities [Table 3] as described in the literature [25, 26, 52, 53], although there was variation the order of performing and number of implementation activities completed. As an example, planning a procedure of how the service would operate in the pharmacy was done by some pharmacies before deciding to adopt the service, as part of exploration stage, whilst the majority of pharmacies completed this activity as part of the preparation stage, after the adoption decision.

Reasons some pharmacies struggled with implementation or moved backwards between stages (such as stopping for a period of time) included, skipping important implementation activities, being deficient in a fundamental influence, or having barriers and lacking strategies to overcome them. For instance although all pharmacies appeared to have a driver for change, such as financial pressure, some lacked communication and teamwork, or top level leadership, which were revealed as vital drivers. Moreover,  whilst in some cases not all activities were required, in other cases activities were missed at the detriment of successful implementation. Interestingly, a trend was seen that those pharmacies who considered more activities were more advanced in implementation, either by number of services being provided (reach) or the perceived integration of service into practice. In agreement with implementation literature, it therefore appears that the implementation activities and influences are complementary and integrative, where strength in one area may counter-balance a weakness in another [24]. It would be recommended for those wishing to implement to consider the feasibility of each activity and then concentrate on the pharmacy’s strengths, to overcome barriers in the implementation process.

Pharmacies were providing a range of services. Services included those focussing on medicines, such as MedsCheck, services focussed on monitoring, as well as services directed towards a more healthy population, such as screening and health promotions. The analysis did not distinguish between services and it may be possible that different services would require distinct implementation considerations. On the other hand, it appeared that the implementation process and influences were often similar, regardless of service. For example across a number of services it was found pharmacies “struggled to maintain patient demand”. Demand may be influenced by multiple factors including: lack of stakeholder involvement, particularly during the service development stage, lack of pharmacy team involvement and buy-in, poor leadership at system and/or pharmacy level, low awareness or a perception of pharmacy at a local level that is at odds with service provision. Co-design, that necessitates stakeholder contribution, should be considered for the development of future professional pharmacy services.

Pharmacies receiving service support, from being part of a pharmacy group, appeared advantaged compared to those working independently. This appeared to be the case particularly for government funded services and services developed across a group. Generally such pharmacies emerged more knowledgeable on services available, aware earlier of new services and received assistance in implementation activities including procedure planning, training, and monitoring. On the contrary some factors were not affected by type of pharmacy including having open communication channels between the pharmacy team.

Evaluations and the activities related to monitoring have been important themes in implementation literature, yet like other disciplines [54] were underdeveloped. Outcome evaluation and staff performance monitoring, in terms of performance quality or fidelity, was lacking in all cases. Interestingly, quality assurance although not measured was a topic pharmacists widely discussed. There was concern about the lack of monitoring and auditing and the consequence this has had on funding and the sustainability of the services. Pharmacists largely did not take personal responsibility to address this, but rather awaited policy changes or action from the professional bodies.

### Framework for the implementation of services in pharmacy (FISpH)

**Fig. 2:**
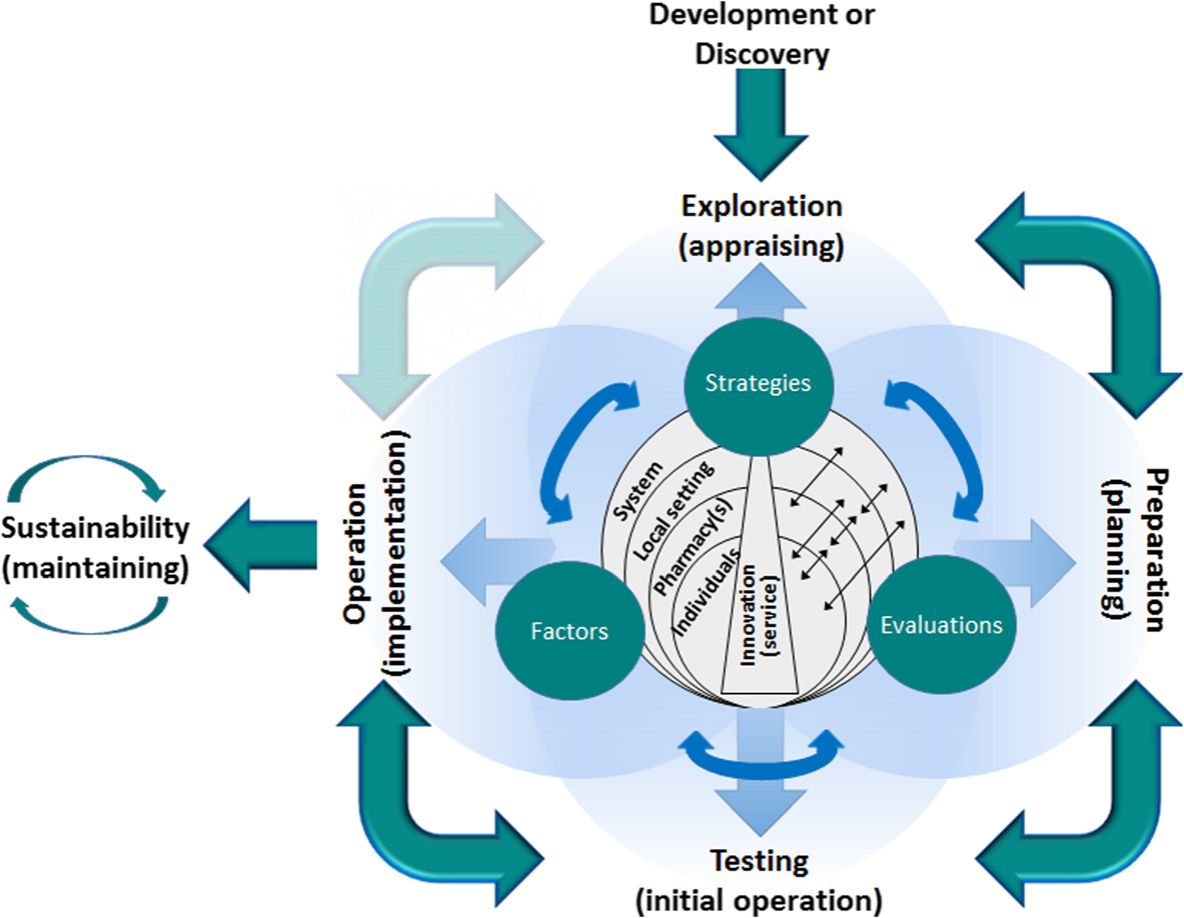
Framework for the Implementation of Services in Pharmacy (FISpH)

**Fig. 3:**
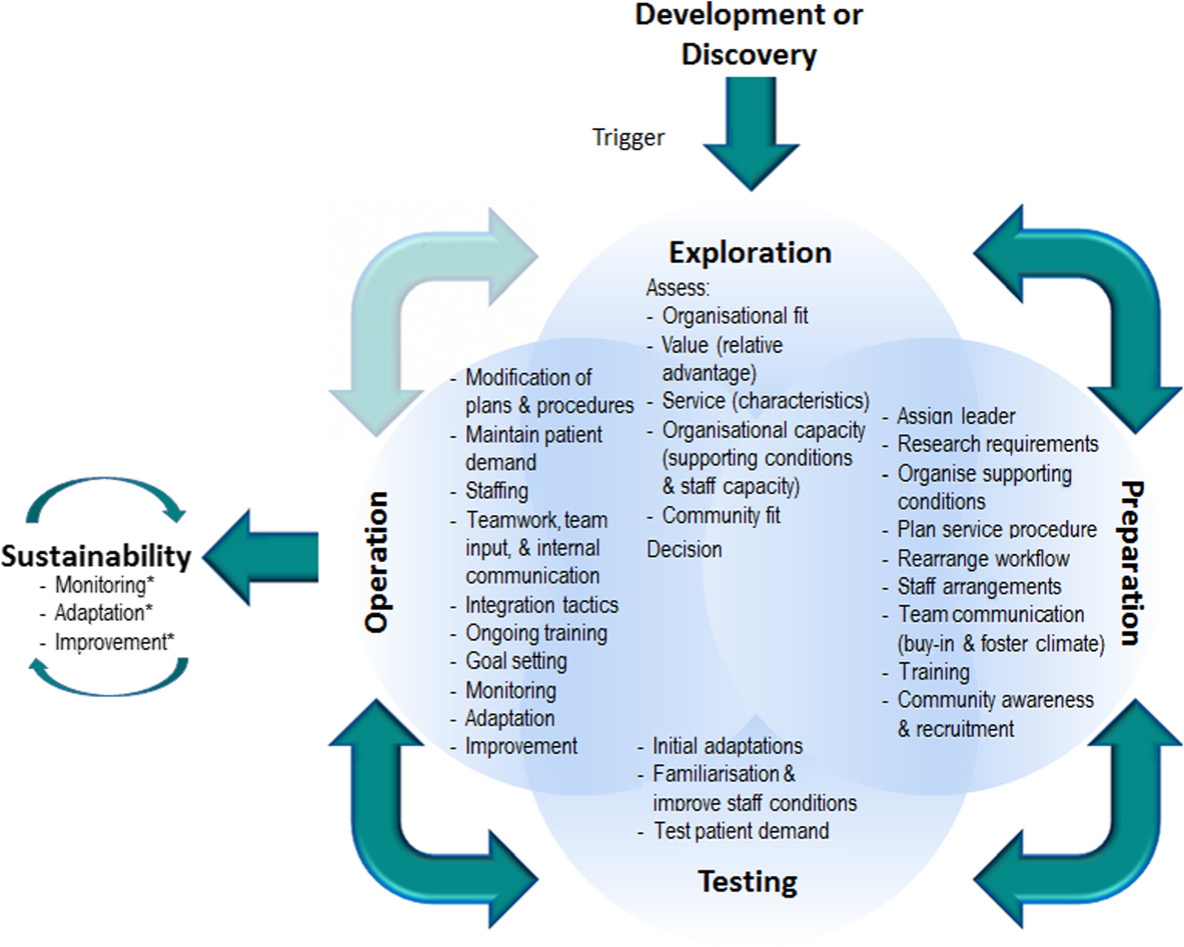
Process of Implementation in Community Pharmacy

The qualitative study has enabled the implementation concepts of the generic implementation framework to be tailored for the implementation of services in pharmacy [Fig. 2]. The data analysis revealed the implementation stages, preceded by development or discovery, as well as the delineation of these stages into a series of activities [Fig. 3]. The data analysis also revealed overarching influences (direction and impetus, internal communication, community fit, staffing and support). A preliminary analysis of the factors that may influence the process at each stage, was also conducted [Additional files 4 and 5], and results may be used as a sub-model for the factors concept of the FISpH. The secondary analysis of factors also verified the domains or ecological levels of influence for implementation professional pharmacy services [20]. Modest investigation of pharmacies utilisation of implementation strategies [Additional file 6] was conducted, but the concept requires further investigation as do implementation evaluations, which were not performed adequately to be studied in detail.

It has been acknowledged there is a lack of theory used in implementation research [2]. This qualitative study provides pharmacy researchers, strategists and practitioners with the foundation of a conceptual framework that may be used as a base for future implementation efforts. The stages and activities may be used to plan implementation programs or protocols, while the influences and list of factors may be used to develop tools, questionnaires or interview guides.

### Considerations for policy, practice and future research

The study revealed that factors vary across the stages and therefore a consideration for practice and future research might be for factor assessments to be conducted at multiple time points, rather than just initially. The adjusted CFIR list has kept the typology and terminology to enable future research and analysis across contexts. The additions require further validation. The list of discrete implementation strategies, along with other change frameworks, may subsequently assist in the selection of suitable approaches, to address or utilise the corresponding factors, both in practice and research projects. To allow implementation strategies to be studied and replicated they must be theoretically derived and reported, as would a clinical intervention [55–57]. It would be recommended that monitoring and evaluation of clinical outcomes, formative evaluations and implementation outcomes be prioritised, by researchers and policy-makers, to facilitate external policy support and the services’ sustainability [58–61].

Subsequent appraisal of the framework depends on its utility, assessed by evaluating programs, that were based on the framework, and if they induced the desired implementation outcome. In other words it is the implementation program that may be validated, which in term evaluates the framework [62].

### Strengths and limitations

Framework analysis showed potential as a methodology for implementation research. In this study the implementation stages were used as overarching themes and thematic analysis performed for the data under each stage. Alternatively interviews could be designed and coded using themes from an implementation factor, strategy or evaluation framework, which would offer interesting insights. A potential limitation of the framework approach is that unless applied in a flexible way, it may inhibit the development or refinement of models. This was prevented by the use of detailed thematic analysis of the activities and influences in addition to the matrix charting. Targeted interview guides based on the meta-frameworks of factors, strategies or evaluations across the stages of the implementation process, could useful for future assessment of these concepts.

A potential source of bias worthy of discussion is effects of a single-coder (JCM) conducting the data collection and analysis [63], although complete consensus has not been reached regarding the use of coding teams [64]. To minimise such affect full thematic and framework analyses were discussed and definitions provided to the co-authors for review with the manuscript. Further studies to confirm and advance the framework and concepts for pharmacy would be recommended.

Purposeful sampling was based on pharmacies level of MedsChecks service provision, while interviews included the exploration other services. As a variation in the degree of implementation was seen during the interviews across the range of services and thematic saturation was achieved across the range of services discussed, no further sampling was deemed necessary. Another sampling limitation is that the study was conducted in Australian pharmacies within two hours from a capital city and 68 % of the interviews in the state of NSW. Although the results are in line with implementation literature they will require further investigation in other states of Australia, rural and remote areas, as well as in other countries. The FISpH however does appears generalizable and understandable by a range of stakeholders, and is currently being used in both Australia and Spain to develop implementation programs and protocols [65].

## Conclusion

The implementation process defined in the literature is largely consistent for implementation in a pharmacy context. The stages and activities of the implementation process appeared as compensatory and did not follow a strict consecutive order, although there was a trend towards the greater the number of activities considered, the greater the integration. Overarching influences were revealed (direction and impetus, internal communication, community fit, staffing and support) and acted as vital drivers to implementation efforts. Improving implementation and service evaluations appeared a critical issue for policy, practice and future research. In addition, future research would be recommended to advance the Framework for the Implementation of Services in Pharmacy (FISpH).

## Additional files

Additional file 1: Implementation definitions. Implementation Science terminology and definitions of the Generic Implementation Framework. (PDF 323 kb) Additional file 2: Interview Guide. Interview guide used to engage pharmacists and facilitate an understanding of Australian pharmacists experiences in the implementation of professional pharmacy services. (PDF 503 kb) Additional file 3: Quotes supporting the implementation process of professional pharmacy services in community pharmacy. Quotes from semi-structured interviews conducted in Australian community pharmacies indicating the process and influences on the implementation of professional pharmacy services. (PDF 340 kb) Additional file 4: List of implementation factors for community pharmacy adjusted from the Consolidated Framework for Implementation Research. List of factors (barriers and facilitators) that may influence the implementation of professional pharmacy services adapted from the Consolidated Framework for Implementation Research and Behavioural Change Wheel. (PDF 68 kb) Additional file 5: Analysis of influencing factors across the stages of implementation. Secondary analysis of factors that appeared in the qualitative study at each stage of the implementation process. (PDF 368 kb) Additional file 6: Analysis of implementation strategies. Secondary analysis of strategies used by pharmacies in the implementation of professional pharmacy services. (PDF 329 kb)

## Abbreviations

BCT: Behavioural change techniques taxonomy
BCW: Behavioural change wheel
CFIR: Consolidated framework for implementation research
EPOC: Cochrane effective practice and organisation of care
FISpH: Framework for the implementation of services in pharmacy
GIF: Generic implementation framework
KPI: Key performance indicator
TDF: Theoretical domains framework
