# Supplementary material for: Qualitative study on the implementation of professional pharmacy services in Australian community pharmacies using framework analysis
Source: BMC Health Serv Res. 2016 Aug 25;16(1):439. doi: 10.1186/s12913-016-1689-7 (PMC4997770; doi:10.1186/s12913-016-1689-7)
Supplement: Additional file 1: — Implementation definitions. Implementation Science terminology and definitions of the Generic Implementation Framework. (PDF 323 kb) [file 12913_2016_1689_MOESM1_ESM.pdf]

Additional File 1: Implementation definitions

**Implementation**	The process of commencing to use and integrating innovations within a setting [1].
**Framework**	Graphical or narrative representation of the key factors, concepts, or variables in order to explain the phenomenon of implementation [2].
**Innovation**	Novel set of behaviours, routines, and ways of working within a setting [3].
**PROCESS OF IMPLEMENTATION**	Non-linear, recursive, reiterative progression of implementation.
**STAGES OF IMPLEMENTATION**	The breakdown of the complete implementation process.
**Development**	Innovation, identification or creation, synthesis, refinement, evaluation and packaging [4].
**Communication**	Process by which people learn and share information about a new innovation to increase awareness [5].
**Diffusion**	Passive, untargeted, unplanned and uncontrolled spread of new innovations [1]. Diffusion is a horizontal or natural process where the onus is on the adopter to seek, absorb and act on the information. Examples of diffusion include mass mailings, publishing in journals and conference presentations. Aim is to increase knowledge and awareness of the innovation.
**Dissemination**	Active approach using planned strategies via determined channels to persuade the target audience to adopt new innovations [1, 3]. Targeted approach takes into account such things as the type of evidence, the end-user(s) needs, and organisational culture and climate. Aim is to increase knowledge, awareness and perception of the innovation.
**Exploration (appraisal)**	The innovation-decision process whereby the end-user(s) appraise the innovation concluding with a decision to either to accept/adopt or reject. Involves progression through awareness (or an issue, need and/or new innovation), knowledge, persuasion, opinion and decision regarding the innovation [5].
**Preparation (planning)**	The course of preparation (innovation, individuals, organization, local environment and external system) prior to innovation use [6].
**Operation (implementation)**	Innovation is in use and is in the process of being integrated into routine practice through active and planned approaches [1].
**Sustainability (maintenance)**	Process of maintaining the innovation through continued innovation use integrated as routine practice, ongoing capacity and supportive environment sufficient to support innovation use and persistence of benefits [7].
**DOMAINS**	Groupings or levels of related implementation influences (and by which factors may be categorised and strategies and evaluations targeted). Domains may vary in number and way in which they are divided.
**Innovation Domain**	A grouping of related influences regarding the characteristics of the innovation to be implemented [8].
**Context Domains**	Groupings of related influences regarding the circumstances that surround the innovation to be implemented [8].
**Individuals**	Characteristics and agency of the people involved with the innovation and/or implementation process.
**Organisation**	Conditions and characteristics of the setting(s) in which the innovation is to operate.
**Local environment**	Circumstances immediately surrounding the organisation(s) including the community, patients and network.
**External system**	Broad economic, political and professional milieu.
**ELEMENTS OF IMPLEMENTATION**	Core considerations affecting the implementation process.
**Factors**	Variables that may affect the implementation process. Also termed facilitators and barriers or determinants of practice [9].
**Strategies**	Targeted efforts (method, technique or activity) designed to enhance moving of an innovation into use and integrating into routine practice [9, 10]. Package of implementation strategies often form an implementation program.
**Evaluations**	Assessment of factors, formative evaluation of strategies, process evaluation and summative evaluation of implementation and innovation outcomes [10-12].

References

1. Rabin BA, Brownson RC, Haire-Joshu D, Kreuter MW, Weaver NL: **A glossary for dissemination and implementation research in health.** *Journal of Public Health Management and Practice* 2008, **14:**117-123.
2. Meyers DC, Durlak JA, Wandersman A: **The quality implementation framework: A synthesis of critical steps in the implementation process.** *American Journal of Community Psychology* 2012, **50:**462-480.
3. Greenhalgh T, Robert G, Macfarlane F, Bate P, Kyriakidou O: **Diffusion of innovations in service organizations: systematic review and recommendations.** *Milbank Quarterly* 2004, **82:**581-629.
4. Graham ID, Logan J, Harrison MB, Straus SE, Tetroe J, Caswell W, Robinson N: **Lost in knowledge translation: Time for a map?** *Journal of Continuing Education in the Health Professions* 2006, **26:**13-24.
5. Rogers EM: *Diffusion of Innovations.* 5 edn. New York: Free Press; 2003.
6. Fixsen D, Naoom, S. F., Blase, D. A., Friedman, R. M., Wallace, F.: **Implementation research: A synthesis of the literature.** In *Book Implementation research: A synthesis of the literature.* (Editor ed.^eds.). City: The National Implementation Research Network, University of South Florida, Louis de la Parte Florida Mental Health Institute.; 2005.
7. Shediac-Rizkallah MC, Bone LR: **Planning for the sustainability of community-based health programs: conceptual frameworks and future directions for research, practice and policy.** *Health Education Research* 1998, **13:**87-108.
8. Damschroder LJ, Aron DC, Keith RE, Kirsh SR, Alexander JA, Lowery JC: **Fostering implementation of health services research findings into practice: a consolidated framework for advancing implementation science.** *Implementation Science* 2009, **4:**50-50.
9. Flottorp S, Oxman A, Krause J, Musila N, Wensing M, Godycki-Cwirko M, Baker R, Eccles M: **A checklist for identifying determinants of practice: A systematic review and synthesis of frameworks and taxonomies of factors that prevent or enable improvements in healthcare professional practice.** *Implementation Science* 2013, **8:**35.
10. Curran GM, Bauer M, Mittman B, Pyne JM, Stetler C: **Effectiveness-implementation hybrid designs: combining elements of clinical effectiveness and implementation research to enhance public health impact.** *Medical Care* 2012, **50:**217-226.
11. Glasgow RE, Vogt TM, Boles SM: **Evaluating the public health impact of health promotion interventions: The RE-AIM framework.** *American Journal of Public Health* 1999, **89:**1322-1327.
12. Proctor E, Silmere H, Raghavan R, Hovmand P, Aarons G, Bunger A, Griffey R, Hensley M: **Outcomes for Implementation Research: Conceptual Distinctions, Measurement Challenges, and Research Agenda.** *Administration and Policy in Mental Health and Mental Health Services Research* 2011, **38:**65-76.
