# Supplementary material for: Qualitative study on the implementation of professional pharmacy services in Australian community pharmacies using framework analysis
Source: BMC Health Serv Res. 2016 Aug 25;16(1):439. doi: 10.1186/s12913-016-1689-7 (PMC4997770; doi:10.1186/s12913-016-1689-7)
Supplement: Additional file 2: — Interview Guide. Interview guide used to engage pharmacists and facilitate an understanding of Australian pharmacists experiences in the implementation of professional pharmacy services. (PDF 503 kb) [file 12913_2016_1689_MOESM2_ESM.pdf]

Additional File 2: Interview Guide

1. Do you currently provide any professional services in your pharmacy?

If planning or providing services:
What would you say is your most successful service, or what service are you planning. Please answer the following questions in regards to this service or services generally.

2. After hearing about the service what drove you to provide the service? [Exploration]
   - *When considering the service what were you looking for?*

3. Do you have any decision process?
   - *How long after hearing about the service did you decide you were going to provide it?*
   - *How did you decide if it is a good idea?*
   - *Did you feel any pressure to adopt services? And if so, from whom?*
   - *Who made the decision to provide the service(s)?*

4. After deciding you wanted to provide a service, what were your next steps? [Preparation]
   - *Did you start providing the service immediately after making the decision to adopt?*
   - *Is someone leading the implementation process? Are all staff involved or particular staff?*
   - *Do you have any specific support for services? (consultants, service manager)*
   - *Have you used, adapted or created any procedures?*
   - *How were you able to accommodate services in your business?*
   - *How did you decide if you were ready to start delivering?*
   - *Any barriers you hit? And how did you overcome them*

5. Once you began delivering the service what did you do in terms of maintaining or improving its provision? [Operation]
   - *Is someone in particular responsible for provision of services?*
   - *How do you identify/recruit patients for services?*
   - *Since commencing have you adjusted the service or workflow in anyway?*
   - *Any barriers you hit? And how did you overcome them?*

6. Do you have any method to evaluate or measure the success of the services you offer? [Operation]
   - *Have you found any unintended benefits or consequences to implementing services?*
   - *Do you use any documentation system?*

7. Would you describe the service provision as routine day-to-day practice? [Sustainability]
   - *What (would) make it routine?*

If you could now think about another service that you do not provide

8. What would cause to decide you want to provide this professional service?

9. After you decided to provide the service what process would you go through or what steps would you to start providing the service?

10. Once the service was being delivered how would you decide whether or not to continue providing the service?

If not providing services:

2. What are your thoughts or opinion about providing professional services?

3. Is there something that would cause you to change your mind and decide you want to provide professional services?

4. After you decided to provide the service what process would you go through or what steps would you to start providing the service?

5. Once the service was being delivered how would you decide whether or not to continue providing the service?
