# Supplementary material for: Qualitative study on the implementation of professional pharmacy services in Australian community pharmacies using framework analysis
Source: BMC Health Serv Res. 2016 Aug 25;16(1):439. doi: 10.1186/s12913-016-1689-7 (PMC4997770; doi:10.1186/s12913-016-1689-7)
Supplement: Additional file 3: — Quotes supporting the implementation process of professional pharmacy services in community pharmacy. Quotes from semi-structured interviews conducted in Australian community pharmacies indicating the process and influences on the implementation of professional pharmacy services. (PDF 340 kb) [file 12913_2016_1689_MOESM3_ESM.pdf]

Additional File 3: Quotes supporting the implementation process of professional pharmacy services in community pharmacy

Exploration

Organisational fit

"If we want to implement that, yes, it is definitely going to be required, yes, it is good for the customer because we focus on customer service and if it is, if we say that we focus on customer service and we don't have this service then what does that mean." (Interview 10)

Value assessment (relative advantage)

"The main goal in the pharmacy is that we are here to help people, to assist people. So as long as the service offers them some sort of health benefit, it will be implemented in the pharmacy. Not only that we do have to consider the business as well. I mean the owners; the services aren't going to pay for themselves. You know, so you have to consider the value of the service to the pharmacy as well." (Interview 5)

Service assessment (service characteristics)

"Has to be easy to follow through on GuildCare [software package] and easy to talk to customers about and doesn't take too long." (Interview 4)

Organisational capacity assessment (supporting conditions & staff capacity)

"*It was a way to get back some funds into the pharmacy, what we were losing through the dispensary, and I thought as a valuable service to the community and to the patients, and something that we could actually implement without too much drama."* (Interview 2)

Community fit assessment

"*We just didn't have the right clientele. We haven't the right socioeconomic for that."* (Interview 20)

"How are going to ever charge for providing a consultation, you know people are so used to walking into the pharmacy and getting it for free." (Interview 8)

Decision (communication, team input and buy-in)

"We work in a way that we seek approval from the team before we roll it out." (Interview 10)

Preparation

Assign leader

"*I would approach the person and ask if they are happy to do it first, and if they are happy then I will change their job description, but they are not happy to do it and they have a reason, I feel that if the person doesn't want to do it there's no point in pushing, so I won't push."* (Interview 10)

"If depends, one of the pharmacy assistants, this is sort of her area. She was in charge of it, so she organised the training for all of us. Something like MedsChecks, which I'm in charge of, I trained all the girls about it. It just depends who is in charge of what." (Interview 22)

"Guess that is where your champion, and you know that gets bandied around a lot, but it is really important to have that person, and for us it is probably our Tech, who then says lets ok, let's get so-and-so, lets book in a few, and start identifying who are the people who are going to do it." (Interview 2)

Research requirements

"*We check to see if we meet the criteria, say a private room or things like that, we want to make sure that we have the resources and everything."* (Interview 10)

"We assign someone, we give a week for that pharmacist – we explain to that pharmacist, that pharmacist does the research and gets back to us with all the requirements, all the information and things like that" (Interview 10)

"Make sure we know exactly what paperwork, what procedures need to be done" (Interview 12)

Organise supporting conditions

"First is, you need to actually get your resources up and running. Whatever resources…so what actually is required to implement it, whether it is equipment, software." (Interview 9)

"We got the program and everything sorted and also the registration with Medicare as well." (Interview 14)

"I had to have my pharmacy redone, so I've got a consulting room where I can provide that sort of service in the right environment" (Interview 20)

Plan service procedure

"Developing the procedure helped me to sort of understand how I was going to approach this otherwise I'd have no idea how it was going to work. And I think that was the main thing, the procedure, having that procedure, writing out what I think should be done, at what point it should be done, who should be involved." (Interview 8)

"We stick to the guidelines, but have our own procedures" (Interview 10)

"Because we are in the city, we needed to do it in a lunch break. Because it really is the only time people would be able to or would be convenient for them to come in" (Interview 1)

"We have a work calendar, and then every month we have a specific topic to focus on and then anything else we just bring that in as well" (Interview 11)

"[Pharmacy group] is really good in that they write up the whole protocol for you already" (Interview 22)

Rearrange workflow

"And because of all these new initiatives, that for us we actually had to change the dispensary flow of things. So for a medium size pharmacy it's challenging because you don't… the pharmacist has to go back and forth a lot." (Interview 25)

Staff arrangements

"We had to get all our timing schedule, I've employed another pharmacist so we've got two pharmacists here now, so before I didn't have the flexibility of senior pharmacists, it's impossible, it's just impossible to do these sort to things" (Interview 20)

"You need to choose a person who is comfortable in doing that. That helps. If you've got someone who is sort of says 'yeah I'll do that,' compared to, there are people where it is out of their comfort zone a little bit." (Interview 2)

"It was mainly employing the right people, who will actually fit in the team, being a good core team…I think before we were so desperate for staff, the previous manager hired sort of anyone" (Interview 22)

"Not exactly new staff, but changing the roles of current staff. So for example, we have a few girls that went from shop floor to dispensary to help dispense so that more pharmacists can get out, get out of the dispensary and talk to patients." (Interview 5)

Team communication (buy-in and foster climate)

"You have to communicate what you're doing so people, when they go up the front counter and go what's happening back there, they need to understand what's happening. We do have more specific, we'll have dispensary meetings and sometimes in dispensary, for certain things, but then we'll bring everyone in on the full staff meetings" (Interview 6)

"So basically everyone – we sort of got together like on a Thursday afternoon it might have even been, it was a bit quiet like now and said how are we going to do this? Get some ideas. Have a think about it. Come back to us and we will go from there. And basically that's how we worked it out." (Interview 8)

"We have poor communication within our staff." (Interview 1)

Training

"An evening with the staff where we can just get… they can get a fundamental knowledge of it" (Interview 19)

"We make sure we're all trained for it and then we implement that training and then we reinforce everyone's knowledge on it, so we have staff meetings to make sure everyone knows what they're doing. And if you know one or two people feel like they're not comfortable with doing it then we'll go through it with them another time and just make sure everyone knows." (Interview 5)

"So what happen is when we go to training... I haven't been to the pain yet, because that's a new one. But with all this training is of course they were explaining to the novel basic theory definitely, so why we want to do it and what benefits it will bring to the patient." (Interview 21)

"The main thing is the training, so we've got [pharmacy group] rep who came around. They do a bit of online training too" (Interview 15)

"We made sure everyone of us knew what was involved in that service. We had a lady [software provider] come and show us and demonstrate what to do."(Interview 5)

Community awareness & recruitment

"Getting the word out, for the patients, it was a bit of a challenge but… sometimes you verbally say, like tell them but it doesn't happen that often, but we have a lot of pamphlets." (Interview 18)

"We've contacted the council, sent out letters to local businesses, things like that." (Interview 25)

"And then normally it depends what sort of program, we might have to like have to say, look out for these sort of patients that's coming through because they will be the ones tied at this program –recruit people." (Interview 14)

"They'll [pharmacy group] start pushing it through catalogues" (Interview 16)

<u>Testing</u>

Initial adaptations

"What I do is I give it a go. I just give it a couple of goes to see what happens. Kind of muddle. I like to give it a go before I can... put anything on the pharmacist. I prefer to kind of take it, and then at least I know what I'm talking about, and people can't say you fobbed it off on me…So I'd probably give it a go, have a bit of a read of the literature, what you have to do and what you have to achieve it in, and then I'd give it a go, see what works, what doesn't work" (Interview 6)

Familiarisation & improve staff conviction

"After the first two that that I'd seen, and then, they were probably our hardest, so after that, we so when we decided we were going to do it" (Interview 1)

Test patient demand

"We usually test it for three months at the start. If customers' not interested we usually then we usually find out to what is sort of required, and then after a few months we will look at rolling out again to see how it goes." (Interview 10)

<u>Operation</u>

Modification of plans & procedures

"We've got like a communal timetable like when to book in the patients. Like it's a very small window, like for example, with Tuesday 10 o'clock to 2:30 only. The little window that we can book them in, that's when the two pharmacists are on duty." (Interview 22)

"Part of our process in terms of you know the dispensary, so if you notice that they need a MedChecks and they fit the criteria you know four or five medications, haven't had one in the last year then yeah, everyone gets a MedChecks. If they approve. But everyone is offered that MedChecks. So it's just part of our dispensing procedure, so in terms of adapting it in to our thing." (Interview 7)

"It´s just a matter of finding the time for it and doing it properly. You don't want to do jobs half-way, you want to do it properly." (Interview 12)

"Because of all these new initiatives, that for use we actually had to change the dispensary flow of things." (Interview 25)

Maintaining patient demand

"We discuss that amongst ourselves as well and so far we've found, you know, certain approaches work on some people and others don't. Some don't… some of our patients don't quite get it at first, do you know what I mean and that sort of thing. So we've tried bags, leaflets, we've tried… we've tried all of that and none of it seems to have worked that well. We have found actually face to face has probably been the best and inviting." (Interview 18)

"A little bit of oversight just trying to figure out how to approach people. That was probably the slowest thing but once we sort of figured out how we were all going to ask the same way." (Interview 4)

"Yeah so we just use the GuildCare program, it will pop up and we can check when they had it last and then we can approach them again." (Interview 3)

Staffing

"Again the accredited pharmacist, she was quite happy, she was quite keen to do it type of thing, although she wasn't quite keen on the sales, trying to get people in thing. But once she was there she was fine. Others just didn't feel comfortable, they... I suppose they didn't feel their knowledge base was strong enough, it was hard to kind of find what the barrier was. And that was... we were kind of in the process of working through those" (Interview 6)

"There was one month where I was tired of… it's like being a salesperson in a way and we didn't do any that month and then afterwards I was like 'OK, I've got to do this.'"(Interview 18)

Teamwork, team input and internal communication

"It is important to have someone who can sell. And as pharmacists ourselves, as pharmacists, we are probably all that good at doing that. So if you've got a Tech or somebody who can do that, I think they're the person to actually do it. Cos as pharmacists we tend to answer questions and give information, but under-sell ourselves if anything. So I think it important if you can identify somebody to/can, and when I say to sell, to actually get the customer or the patient to commit" (Interview 2)

"We all work together, because if you have one pharmacist doing one particular service and that's all that they do and they don't do any other service it kind of traps them in that role. So it's better I think in the pharmacy that we actually have each pharmacist running different types of services, keeps your mind going, engages the patient. You don't get bored doing just that one thing and yeah you have, you know you can practise your different skill sets along all the other different types of services that are available." (Interview 5)

"Yeah depends on the, most of the services there has at least part involvement, obviously the assistants cannot do the MedsCheck but they do know what is everything, what it is about…so when the customer asks they'll know what is it but they'll get someone else." (Interview 24)

Integration tactics

"We just would, like constantly remind them yeah…You just ask them just constantly, every single checking script "Did they qualify? Did they qualify? Did they qualify?" and they'd have to double check it again." (Interview 3)

"If you don't have it at the top of your priority on the list of your tasks, you will spend the whole day and won't even think of it once." (Interview 9)

"We have a system where they will readily have it in their pocket and it's something that is triggers them." (Interview 10)

"We have this note, OK. We put it in every basket. So we have all the programs that we provide. OK. So it's just as a reminder for us, pharmacist and the customers really." (Interview 21)

"You've got to have some sort of lead in to it, if you know what I mean? I mean in our case we've got the Guild Care program, which gives you a lot of... it flashes at you when the person has done five medications, or it'll tell you... it'll flash at you if somebody is on a puffer and they need." (Interview 16)

"We've just implemented the health screening, it's not… I mean I haven't actually screened anyone because it's kept in a file and it's kept in the drawer, and you don't remember it." (Interview 25)

"So accountability. So at the end of every week I wanted the report emailed. So that was even messed up a couple of times. So I was like just as you get paid every week with your weekly roster you need to send me a weekly account of how many clinical interventions you are doing." (Interview 9)

"Mandating it as part of their performance reviews. To make sure they actually do" (Interview 7)

Ongoing training

"Then we reinforce everyone's knowledge on it, so we have staff meetings to make sure everyone knows what they're doing." (Interview 5)

"We just find our own examples. Say for example if something that I come across with the customers I would then use it as an example to tell the team that that is a clinical intervention." (Interview 10)

"I just go the GuildCare lady coming in and showing the interns how to do them again." (Interview 4)

Goal setting

"You have to get the message right to everyone…and then we set a weekly team target…so I guess as we break it down it seems like less, you know easier for the staff as well to focus on." (Interview 11)

"KPIs are a good thing to be able to be able to share with staff, and I think that, and the staff do they understand that we get paid for these and they understand that this contributes towards their wages" (Interview 2)

"With my other pharmacist I've said to him I want you to do one a week, OK, I want you to really try to do one a week. So I put a bit of pressure on him to do one a week, and I said I'll do one as well. And if we can do that I think that's a good start. I think if you start doing it right then it starts to build and it becomes routine." (Interview 20)

"Only by physically doing them myself did I realise what is an achievable goal. People will actually say to you, I'm too busy, oh I can't pick up an intervention, you can hear a lot of reasons why it is not being implemented within your store. But you need to actually to have the grass roots yourself, know if you goals are set too high or you have goals that are achievable or not achievable." (Interview 9)

Monitoring

"So clinical interventions [type of service] are the same, we are rolling out well and then there will be a time where the team will go a bit down and then we will see the numbers drop because we know at least it will be that. Say for example we have 200 customers a day – I would say that as a minimum I would have clinical intervention would be 5% - really at least five customers that we would have certain intervention, but if anything that is dropping below that it triggers me to say hey it's time to perhaps realign the team again or we do the training again" (Interview 10)

"As far as monitoring we do look at how many we are doing, how many we are doing on a monthly basis and whatever and if we are not doing enough consistently than we talk about well how we are going to get them going again." (Interview 2)

"The data is from all the branches. It's not like from the sessions that you have in one branch, it's all branches, and then they collect like the data." (Interview 21)

"If anything we would probably look at on those days when we do have a health service how are we going with sales in store and if sales increase you have got a pretty good indication that it's because you have got something going on to get the customers in." (Interview 12)

"We will still take feedback as we go and then make it better and better" (Interview 11)

"A few negative feedback won't stop us from stopping the service altogether. It is more a bit of say after we've done it and then come back and found OK this is how much time we spent on it, this is what we got out of it, like and then we decide whether it was worthwhile to do it. Sometimes we don't do it like all year – we just choose a few months in the year to do it." (Interview 11)

"The guidelines always say it's between 20-30 minutes, but sometimes it can drag on a bit longer, so that is the thing…Usually if I see something like that I would sit down with the Pharmacist and say, look it has been taking a bit longer than the time we can assign for what we can do now." (Interview 10)

"You know what I base it on? The effect it has on my patients, do you know what I mean, in terms of how much they thought it was of value to them, and you normally can tell by the end of the thing. Because of the feedback that you get from them, do you know what I mean? And also then the follow up when I see them again" (Interview 20)

Adaptation

"It was theoretical, it didn't work in practice with the appointments, with consent forms." (Interview 3)

"When we first started approaching people on the spot we would only get maybe two out of ten who were happy to actually do it straight away, so we thought how can we go about this another way and that is how we thought of it we do it bit by bit by only taking up five or ten minutes of their time each time, then they're generally happy to do it that way." (Interview 6)

"And then give and take, is it working, if it's not we've got to change something. So like MedsCheck is not really working for us, so we've got to try and get a formula for instigating it, do you know what I mean?" (Interview 18)

"MedsCheck and diabetes I rarely do appointments, because people don't want to come back for it, I usually try and get it on the spot if I can." (Interview 18)

Improvement

"Yeah, and feedback. It's just, it's little things like we should have all the paperwork printed off ready so that we don't have to go and do that afterwards, do you know what I mean, the claiming sheets and all that sort of things, so we can do it all in one nice unit, and then it's done and we can fax it off and things like that. So that sort of thing. Printing out all their background

before they come in, so we've got it all ready, we've looked at it we're a bit familiar with it, and then so that when they come it's correct" (Interview 20)

"You get more comfortable in the speech of selling it, you become more comfortable with the programs you are using, the amount of time they take becomes shorter, so more manageable I guess. Don't get me wrong, some take a longer time, and some a shorter time." (Interview 2)

"Once you get it builds into a more routine, much more streamlined. And so we have got the paperwork ready, we've got everything ready to go, so it's not a big deal" (Interview 20)

"Basically just more of like a teamwork, and more procedure wise and make sure everyone was up to date on how everything worked and how everything is meant to be done, so rather than having one person do something one way and one another way, everyone is on the same page." (Interview 8)

Sustainability

"It became everyday thing now. It's more routine…that's why we don't really separate that. It's like a routine that we make sure everybody does." (Interview 10)

""The MedChecks was getting to be part of routine practice, and we've all used it, and people are accepting of it. We had done, some of our database the first year, and we're kind of on to our second year of doing them, and we're increasing our tool with it, like new diagnosis, new patients, and things like that. People that really need to be told well what's happening here." (Interview 6)

"It's not new. So what happen is like, things like type 2 diabetes program, we had training since 2012 I think, when they start wanting to do this, and they put in a system how to do it. It's from 2012. And at that time, because I wasn't in this store yet, I was in bigger store, and at the time we do it for free. But then now there's a charge for it." (Interview 21)

"Well the thing is, you almost have to do a shorter time frame to access more people, because if you're going to make a difference to your practice you're going to need to... you know it's alright to… what´s my analogy, you can hug one person, but if you're better off having a handshake with ten people, and get more people involved so they can actually get exposed to it. Otherwise you don't actually make a difference. You can't just do it to a small selective, you've got to get a physical, critical mass to get through. So maybe you've got to bring it down to 15 minutes. And do more people in a day." (Interview 6)

"Yeah we are kept going. I mean the main aim of MedChecks is to help patients. And you're not going to stop helping patients because you're not going to be paid for that any more. I mean it's harder to afford to have the amount of pharmacists that we do without getting that funding but you know there are ways about it. You have to just be smart about it, conduct it in ways that are quicker, be more efficient about it, make sure patients are engaged in their you know they know what they're doing and they understand their medications." (Interview 5)
