# Supplementary material for: Qualitative study on the implementation of professional pharmacy services in Australian community pharmacies using framework analysis
Source: BMC Health Serv Res. 2016 Aug 25;16(1):439. doi: 10.1186/s12913-016-1689-7 (PMC4997770; doi:10.1186/s12913-016-1689-7)
Supplement: Additional file 4: — List of implementation factors for community pharmacy adjusted from the Consolidated Framework for Implementation Research. List of factors (barriers and facilitators) that may influence the implementation of professional pharmacy services adapted from the Consolidated Framework for Implementation Research and Behavioural Change Wheel. (PDF 68 kb) [file 12913_2016_1689_MOESM4_ESM.pdf]

Additional File 4: List of implementation factors for community pharmacy adjusted from the Consolidated Framework for Implementation Research [1]

Domains & Factors

	**INNOVATION (SERVICE) (b)**	**Definition**
1	Source (a)	Perception of key stakeholders about whether the innovation is externally (for example by a professional body, university, pharmaceutical company, or government) internally (individual pharmacy or pharmacy group) developed (b) [1]
2	Evidence strength & quality (a)	Stakeholders' perceptions of the quality and validity of evidence supporting the belief that the innovation will have desired outcomes (a) [1]
3	Relative advantage (a)	Stakeholders' perception of the advantage or value of the innovation versus an alternative solution (b, e) [1]
	3a. Direct financial benefits (e)	Direct financial compensation for example from government, company or patient
	3b. Other organisational benefits (e)	Business benefits as a result of the innovation such as increasing patient loyalty, return rates, community rapport, sales, efficiency etc.
	3c. Patient benefits (e)	Improved patient outcomes such as health, quality of life, adherence, knowledge confidence etc.
	3d. Professional/personal benefits (e)	Professional or personal reward such as increased satisfaction or motivation
4	Adaptability (a)	The degree to which an innovation can be adapted, tailored, refined, or reinvented to meet local needs (a) [1]
5	Trialability (a)	The ability to test the innovation on a small scale in the organization, and to be able to reverse course (undo implementation) if warranted (a) [1]
6	Implementation complexity (b)	Perceived difficulty of implementation, reflected by duration, scope, radicalness, disruptiveness, centrality, and intricacy and number of steps required to implement (a) [1]
7	Design quality & packaging (a)	Perceived excellence in how the innovation is bundled, presented, and assembled (a) [1]
8	Cost (a)	Costs of the innovation and costs associated with implementing the innovation including investment, supply, and opportunity costs (a) [1]
9	Nature of innovation (e)	Nature of the service in terms of degree of change from previous habit (organisational practices and work routines) including innovation difficulty and extent of other healthcare professionals' involvement (c, e) [2]
10	Duration (e)	Duration of innovation including frequency of follow-up and regularity throughout the year
11	Quality assurance system (e)	Method to assess quality of the innovation's implementation and provision

	**INDIVIDUALS (a)**	
1	General knowledge (c)	Domain or general knowledge acquired from education, training, or accreditations on conditions, pharmacology, scientific rationale or the pharmacy environment and management (c) [2]
2	Knowledge about the innovation (b)	Individuals' comprehension with facts, requirements truths, principles and practices related to the innovation (b) [1]
3	Beliefs about the innovation (b)	Individuals' agreement with the innovation in terms of their attitude towards, value placed and expected outcomes or consequences (b, c) [1,2]
4	Self-efficacy (a)	Individual belief in their own capabilities to execute courses of action to achieve implementation goals (a) [1]
5	Individual state of change (a)	Characterisation of the phase an individual is in, as he or she progresses toward skilled, enthusiastic, and sustained use of the innovation (a) [1]
	5a. Technical skills (experience, capacity & competence) (c)	Familiarity, ability and expertise in performing the tasks involved in innovation provision including interpretation of results (c) [2]
	5b. Interpersonal skills (experience, capacity & competence) (c)	Communication skills and ability to relate and interact with patients, colleagues and other healthcare professionals (c) [2]
6	Individual identification with organisation (a)	A broad construct related to how individuals perceive the organisation and their relationship and degree of commitment with that organisation (a) [1]
7	Other personal attributes (a)	A broad construct to include other personal traits such as tolerance of ambiguity, intellectual ability, learning style, emotions and coping strategies) (b, c) [1,2]
8	Values & motivation (b)	A person's professional identity, satisfaction, and intrinsic motivation which may be portrayed as intention and goals (c) [2]
9	Leadership skills (d)	Ability to inspire and motivate others as well as make sound decisions
10	Memory, attention and decision processes (c)	The ability to remember and retain information, focus selectively on aspects of the environment and choose between two or more alternatives, dedicate which may be lead to cognitive overload, tiredness, time dedicated to the service and its implementation and self-monitoring (c) [2]

	ORGANISATION (PHARMACY(S)) (b)	
1	Structural characteristics (a)	The social architecture, age, maturity, size, script volume and location of an organisation (b) [1]
2	Staff (d)	Sufficient and qualified staff/manpower
3	Layout & workflow (d)	Physical arrangement of the organisational environment
4	Networks & internal communication (b)	The nature and quality of webs of social networks and the nature and quality of formal and informal communications within an organization (a) [1]
5	Teamwork (d)	Combined action of a group each doing their own part to aid effectiveness and efficiency
6	Autonomy (d)	Right to self-regulate, work and make decisions independently
7	Culture and vision (b)	Norms, values, and basic assumptions of a given organization including organisational direction (b) [1]
8	Implementation climate (a)	The absorptive capacity for change, shared receptivity of involved individuals to an innovation and the extent to which use of that innovation will be rewarded, supported, and expected within their organisation (a) [1]
	8a. Tension for change (a)	The degree to which stakeholders perceive the current situation as intolerable or needing change (a) [1]
	8b. Compatibility (a)	The degree of tangible fit between meaning and values attached to the innovation by involved individuals, how those align with individuals' own norms, valued and perceived risks and needs, and how the intervention fits with existing workflows and systems (a) [1]
	8c. Relative priority (a)	Individuals' shared perception of the importance of the implementation within the organization (a) [1]
	8d. Organisational incentives & rewards (a)	Extrinsic incentives such as goal-sharing awards, performance reviews, promotions, and raises in salary and less tangible incentives such as increased stature or respect (a) [1]
	8e. Goal setting (b)	Establishing targets and objectives for the innovation
	8f. Feedback (b)	The degree to which goals are clearly communicated, acted upon, and fed back to staff and alignment of that feedback with goals (a)
	8g. Learning climate (a)	A climate in which: a) leaders express their own fallibility and need for team members' assistance and input; b) team members feel that they are essential, valued, and knowledgeable partners in the change process; c) individuals feel psychologically safe to try new methods; and d) there is sufficient time and space for reflective thinking and evaluation. (a) [1]
9	Readiness for implementation (a)	Tangible and immediate indicators of organizational commitment to its decision to implement an innovation (a) [1]
	9a. Leadership engagement (a)	Commitment, involvement, and accountability of leaders and managers with the implementation (a) [1]
	9b. Available resources & training (a)	The level of resources dedicated for implementation and on-going operations including money, training, education and time (b) [1]
	9c. Access to knowledge & information (a)	Ease of access to digestible information and knowledge about the intervention and how to incorporate it into work tasks (a) [1]
10	Data management system (d)	Recording system for the innovation and information related to its implementation and provision
11	Quality assurance system (c)	Method or activities to assess quality of innovation implementation and/or provision
12	Environmental stressors (c)	Balance between competing demands and/or conflicting roles and available resources, including time
13	Organisational support and/or assistance (e)	Support provided by the organisational group or head office such as advertising, training, monitoring etc.
14	Experience (e)	Degree of observation or participation with the innovation or similar innovations previously

LOCAL SETTING (d)

1	Interprofessional network & communication (b)	The degree to which an organisation is networked and interacts within their profession (b) [1]
2	Intraprofessional network & communication (b)	The relationship, social networks and profile an organisation has with other local healthcare professionals and organisations (b) [1]
3	Community's perception about innovation and organisation (d)	Local population's knowledge, beliefs and expectations regarding the innovation
4	Relationship with patients and community (e)	Profile of the organisation within the community and rapport with their patients
5	Demand (d)	Perception of key stakeholders´ about the level of demand or interest in the innovation including the ease of recruiting patients in the service.
6	Patient needs & resources (a)	The extent to which patient needs, as well as barriers and facilitators to meet those needs are accurately known and prioritised by the organisation (a) [1]
7	Peer Pressure (a)	Mimetic or competitive pressure to implement an innovation; typically because most or other key peer or competing organizations have already implemented (a) [1]

EXTERNAL SYSTEM (b)

1	Laws, policies or regulations (b)	Includes policy and regulations (governmental or other central entity), external mandates, recommendations and guidelines, pay-for-performance, collaboratives, public or benchmark reporting, or accreditation systems (b) [1]
2	Remuneration (b)	Model and degree of funding
3	Healthcare budget & contracts (b)	Payer polices including the duration and stability of contracts
4	Intraprofessional networks & communication (b)	The degree to which the profession is networked with other healthcare professions and their organisations (cosmopolitism) (b) [1]
5	Interprofessional relations & leadership (e)	The degree of consolidarity within the profession and their professional organisations
6	Stakeholder buy-in (e)	Acceptance of service from pharmacy organisations, other healthcare professional organisations and government
7	External support and/or assistance(d)	Support for professional organisations, companies or government in terms of materials, software, guidelines, training

(a) Direct from consolidated framework for implementation research [1] (b) Modified from consolidated framework for implementation research
(c) Adapted from theoretical domains framework [2] (d) Derived from pharmacy practice research [3] (e) Resulting from qualitative thematic analysis of implementation process for professional pharmacy services in community pharmacy

References

1. Damschroder LJ, Aron DC, Keith RE, Kirsh SR, Alexander JA, Lowery JC: **Fostering implementation of health services research findings into practice: a consolidated framework for advancing implementation science**. *Implement Sci* 2009, **4**(1):50-50.
2. Michie S, Atikins L, West R: **The behavioural change wheel: a guide to designing interventions**. Great Britain: Silverback; 2014.
3. Roberts AS, Benrimoj SI, Chen TF, Williams KA, Aslani P: **Implementing cognitive services in community pharmacy: a review of models and frameworks for change**. *Int J Pharm Pract* 2006, **14**(2):105-113.
