# Supplementary material for: Qualitative study on the implementation of professional pharmacy services in Australian community pharmacies using framework analysis
Source: BMC Health Serv Res. 2016 Aug 25;16(1):439. doi: 10.1186/s12913-016-1689-7 (PMC4997770; doi:10.1186/s12913-016-1689-7)
Supplement: Additional file 5: — Analysis of influencing factors across the stages of implementation. Secondary analysis of factors that appeared in the qualitative study at each stage of the implementation process. (PDF 368 kb) [file 12913_2016_1689_MOESM5_ESM.pdf]

Additional File 5: Analysis of influencing factors across the stages of implementation

	INNOVATION (SERVICE)	Development or discovery	Exploration	Preparation	Testing	Operation	Sustainability
1	Source	X	X				X
2	Evidence strength & quality		X			X	
3	Relative advantage	X	X		X	X	X
	3a. Direct financial benefits		X			X	X
	3b. Other organisational benefits		X		X		
	3c. Patient benefits		X		X		
	3d. Professional/personal benefits		X			X	
4	Adaptability				X	X	X
5	Trialability			X	X		
6	Implementation complexity		X	X	X	X	
7	Design quality & packaging		X	X	X	X	
8	Cost		X	X		X	
9	Nature of innovation		X		X	X	
10	Duration		X	X	X	X	
11	Quality assurance system		X	X		X	X

	INDIVIDUALS	Development or discovery	Exploration	Preparation	Testing	Operation	Sustainability
1	General knowledge	X	X	X	X	X	X
2	Knowledge about innovation	X	X	X	X	X	
3	Beliefs regarding innovation		X	X	X	X	X
4	Self-efficacy		X		X	X	
5	Individual state of change		X	X	X	X	X
	5a. Technical skills (experience, capacity & competence)		X		X	X	
	5b. Interpersonal skills (experience, capacity & competence)			X	X	X	
6	Identification with organisation					X	X
7	Other personal attributes	X		X		X	
8	Values & motivation		X		X	X	X
9	Leadership skills		X	X	X	X	X
10	Memory, attention & decision processes				X	X	

	ORGANISATION (PHARMACY(S))	Development or discovery	Exploration	Preparation	Testing	Operation	Sustainability
1	Structural characteristics	X	X	X		X	
2	Staff	X	X	X	X	X	X
3	Layout & workflow		X	X	X	X	
4	Networks & internal communication	X	X	X	X	X	X
5	Teamwork		X	X	X	X	
6	Autonomy	X	X		X	X	
7	Culture and vision	X	X	X	X	X	X
8	Implementation climate	X	X	X	X	X	
	8a. Tension for change	X	X	X		X	X
	8b. Compatibility		X		X	X	
	8c. Relative priority		X	X	X	X	
	8d. Organisational incentives & rewards		X		X	X	
	8e. Goal setting			X	X	X	
	8f. Feedback				X	X	
	8g. Learning climate		X	X	X	X	
9	Readiness for implementation		X	X	X	X	
	9a. Leadership engagement	X	X	X	X	X	X
	9b. Available resources		X	X	X	X	
	9c. Access to knowledge & information			X		X	
10	Data management system		X	X	X	X	X
11	Quality assurance system		X			X	X
12	Environmental stressors		X		X	X	
13	Organisational support and/or assistance	X	X	X		X	X
14	Experience		X		X	X	

	LOCAL SETTING	Development or discovery	Exploration	Preparation	Testing	Full operation	Sustainability
1	Interprofessional network & communication	X	X	X			
2	Intraprofessional network & communication			X	X	X	X
3	Community's perception about innovation and organisation	X	X	X	X	X	X
4	Relationship with patients and community		X		X	X	X
5	Demand	X	X	X	X	X	X
6	Patient needs & resources	X	X	X	X	X	X
7	Peer Pressure	X	X				X

	EXTERNAL SYSTEM						
1	Laws, policies or regulations	X		X			X
2	Remuneration		X		X	X	X
3	Healthcare budget & contracts		X			X	X
4	Intraprofessional networks & communication						X
5	Interprofessional relations & leadership			X			X
6	Stakeholder buy-in	X				X	X
7	External support and/or assistance	X	X	X	X	X	
