# Supplementary material for: Qualitative study on the implementation of professional pharmacy services in Australian community pharmacies using framework analysis
Source: BMC Health Serv Res. 2016 Aug 25;16(1):439. doi: 10.1186/s12913-016-1689-7 (PMC4997770; doi:10.1186/s12913-016-1689-7)
Supplement: Additional file 6: — Analysis of implementation strategies. Secondary analysis of strategies used by pharmacies in the implementation of professional pharmacy services. (PDF 329 kb) [file 12913_2016_1689_MOESM6_ESM.pdf]

Additional File 6: Analysis of implementation strategies

	Strategy [1]	Utilised	Not-utilised
1	Access new funding	X	
2	Alter incentive/allowance structures	X	
3	Alter patient/consumer fees	X	
4	Assess for readiness and identify barriers and facilitators	X	
5	Audit and provide feedback	X	
6	Build a coalition	X	
7	Capture and share local knowledge	X	
8	Centralize technical assistance	X	
9	Change accreditation or membership requirements	X	
10	Change liability laws		X
11	Change physical structure and equipment	X	
12	Change record systems	X	
13	Change service sites	X	
14	Conduct cyclical small tests of change	X	
15	Conduct educational meetings	X	
16	Conduct educational outreach visits	X	
17	Conduct local consensus discussions		X
18	Conduct local needs assessment	X	
19	Conduct ongoing training	X	
20	Create a learning collaborative	X	
21	Create new clinical teams	X	
22	Create or change credentialing and/or licensure standards	X	
23	Develop a formal implementation blueprint	X	
24	Develop academic partnerships	X	
25	Develop an implementation glossary		X
26	Develop and implement tools for quality monitoring		X
27	Develop and organize quality monitoring systems		X
28	Develop disincentives	X	
29	Develop educational materials	X	
30	Develop resource sharing agreements	X	
31	Distribute educational materials	X	
32	Facilitate relay of clinical data to providers	X	
33	Facilitation	X	
34	Fund and contract for the clinical innovation	X	
35	Identify and prepare champions	X	
36	Identify early adopters		X
37	Increase demand	X	
38	Inform local opinion leaders		X
39	Intervene with patients/consumers to enhance uptake and adherence	X	
40	Involve executive boards		X
41	Involve patients/consumers and family members		X

42	Make billing easier		X
43	Make training dynamic		X
44	Mandate change	X	
45	Model and simulate change	X	
46	Obtain and use patients/consumers and family feedback	X	
47	Obtain formal commitments		X
48	Organize clinician implementation team meetings	X	
49	Place innovation on fee for service lists/formularies	X	
50	Prepare patients/consumers to be active participants		X
51	Promote adaptability	X	
52	Promote network weaving	X	
53	Provide clinical supervision		X
54	Provide local technical assistance	X	
55	Provide ongoing consultation		X
56	Purposely reexamine the implementation		X
57	Recruit, designate, and train for leadership	X	
58	Remind clinicians	X	
59	Revise professional roles	X	
60	Shadow other experts	X	
61	Stage implementation scale up	X	
62	Start a dissemination organization		X
63	Tailor strategies	X	
64	Use advisory boards and workgroups		X
65	Use an implementation advisor		X
66	Use capitated payments	X	
67	Use data experts		X
68	Use data warehousing techniques	X	
69	Use mass media	X	
70	Use other payment schemes		X
71	Use train-the-trainer strategies	X	
72	Visit other sites	X	
73	Work with educational institutions	X	

Reference

1. Powell B, Waltz T, Chinman M, et al. A refined compilation of implementation strategies: results from the Expert Recommendations for Implementing Change (ERIC) project. *Implement Sci.* 2015;10(1):21.
